# Comorbid Conditions in Kidney Transplantation: Outcome Analysis at King Abdulaziz Medical City

**DOI:** 10.7759/cureus.41355

**Published:** 2023-07-04

**Authors:** Abdulrahman R Al Tamimi, Bader A Aljaafri, Fahad Alhamad, Sultan Alhoshan, Awatif Rashidi, Basayel Dawsari, Ziad A Aljaafri

**Affiliations:** 1 Hepatobiliary Sciences and Organ Transplantation, King Abdulaziz Medical City, Riyadh, SAU; 2 College of Medicine, King Saud Bin Abdulaziz University for Health Sciences, Riyadh, SAU; 3 Biostatistics, King Abdullah International Medical Research Center, Riyadh, SAU

**Keywords:** comorbid disease, outcomes and complication of renal transplantation, post transplantation, outcome, kidney transplantation

## Abstract

Background: Kidney transplantation is most commonly performed for end-stage renal disease (ESRD) and provides the best chance for a cure. The surgery is shown to be beneficial to a patient’s quality of life after transplantation in multiple studies. But graft failure is a serious consequence that might happen. The term graft failure refers to the failure of a transplanted kidney to function properly. There are various reasons why this can happen, such as rejection, infection, or medication complications.

Methods: A retrospective cohort study of comorbid conditions in patients who underwent renal transplantation at King Abdulaziz Medical City (KAMC) between 2016 and 2022. Data were collected by chart review using the BestCare system. The data collected included patients’ demographics, comorbidities, calculated Charlson Comorbidity Index (CCI), surgery-related data, laboratory data, and the outcome of transplantation. The categorical data were presented using percentages and frequencies, while the numerical data were presented as mean and standard deviation. The Chi-square test was used for inferential statistics to find the association between categorical variables.

Results: A total of 669 patients were included in the current study. Of these, 422 (63.1%) were men, and the mean age was 44 years. The incidence of graft failure within one year at KAMC was found to be 1.2% (eight cases). Regarding the CCI and its association with graft failure within one year, 37 (5.5%) patients had a myocardial infarction (MI) and 17 (2.5%) had congestive heart failure; however, no patients with MI or congestive heart failure experienced graft failure, and no significant association was found between MI or congestive heart failure and graft failure (p-value = 1.000 for both). A total of 417 (62.3%) patients had no or diet-controlled diabetes, 122 (18.2%) had uncomplicated diabetes mellitus (DM), and 130 (19.4%) had end-organ damage. DM and graft failure were not significantly associated (p-value = 1.000). A total of 286 (42.8%) patients had ESRD of unknown etiology, 109 (16.3%) patients had ESRD caused by diabetic nephropathy, and 100 (14.9%) had ESRD resulting from hypertension, apart from other causes.

Conclusion: Most patients were found to have ESRD of unknown etiology and the most frequently reported known risk factor for ESRD and subsequent transplantation was found to be diabetic nephropathy, followed by hypertension.

## Introduction

Kidney transplantation is the definitive treatment for end-stage renal disease (ESRD) and is considered the best option for patients on dialysis [1-3]. There are two approaches to kidney transplant, namely laparoscopy and open surgery [1]. Multiple studies have reported the benefits of this surgery on patients’ quality of life after transplantation [1,4]. However, comorbidities such as diabetes and heart failure can influence the outcome of the surgery. Researchers reported that an increased number of comorbidities both before and after transplantation increased the chance of death [5]. In another study, renal failure and hypertension (HTN) were found to be the most common etiologies of ESRD [6].

Patients who reach stage 4 of chronic kidney disease (CKD), defined as a glomerular filtration rate (GFR) of less than 30 mL/min/1.73 m^2^, should seek medical help and be informed regarding kidney failure and possible treatments, including transplantation [7]. Comorbidities affect perioperative and long-term outcomes as well [5].

Multiple indices are used to evaluate the effect of comorbidities on ESRD patients [8], including the Index of Coexistent Disease (ICED) and Khan, Davies, and Charlson scores, all developed and applied to the ESRD population [5,9-11]. The Charlson Comorbidity Index (CCI) is a valuable tool for assessing comorbidity in transplant patients [5]. A study found that CCI is the most sensitive in discriminating features, with a concordance C statistic of 0.71 [8]. Moreover, CCI is the most used score to measure comorbidity [12].

Even though many studies have reported the effect of multiple comorbidities on patients with ESRD, research on their impact on kidney transplant outcomes is insufficient. As the number of studies assessing the effects of comorbid conditions on kidney transplant patients is limited, we decided to evaluate the comorbidities in patients who underwent kidney transplant at King Abdulaziz Medical City (KAMC), Riyadh, Saudi Arabia.

## Materials and methods

This retrospective cohort study was conducted at KAMC, Riyadh, Saudi Arabia. We included all patients who underwent kidney transplantation between 2016 and 2022. The sampling technique utilized for the study was non-probability consecutive sampling, including all patients who met the inclusion criteria. Data were collected by the research team members by chart review using the BestCare system at KAMC. The data collected included patients' data, comorbidities, surgery details, donor characteristics, outcome variables, and grouping variables. Comorbidities include myocardial infarction (MI), congestive heart failure, peripheral vascular disease, cerebrovascular disease, dementia, chronic obstructive pulmonary disease (COPD), connective tissue disease, peptic ulcer disease (PUD), liver disease, diabetes mellitus (DM), solid tumor, depression, arthritis, asthma, and hypertension. We also collected HCV antibody results for patients. Also estimated GFR (eGFR) and creatinine were gathered to compare values before and after transplantation. After the transplant values were collected on day 0, day 1, day 7, day 30, day 60, day 90, and day 180.

The CCI was used to assess different comorbidities. CCI is a commonly used method for predicting mortality risk in those who have chronic health conditions [5]. The CCI has been used in a wide range of clinical settings. It has been shown to be helpful as a tool for predicting outcomes in a variety of patient populations, including those with cancer, heart disease, and CKD [9-11].

For data management and analysis, SPSS version 23 (IBM Corporation, Armonk, NY, USA) was used. Mean and standard deviation were used for quantitative variables, while qualitative variables were expressed as percentages and frequencies. The normality of the data was checked using the K-S test. The chi-square test, t-test, and nonparametric tests were used to compare qualitative and quantitative variables between groups. A p-value of 0.05 was considered significant.

Ethical approval was obtained from the Institutional Review Board to access the medical records. All the information was accessed only by the principal investigator and co-investigators of the study. The researchers ensured subjects’ privacy and confidentiality by not collecting any identifiers such as medical record number (MRN), names, and identification. All data were securely stored within KAMC premises, and access to the research data was restricted to the research team members.

## Results

A total of 669 patients were included in the current study. Their mean age was 44.4 ± 17 years (1-86 years). The mean age of those with graft failure was 43.6 ± 19.9 years. The mean body mass index (BMI) was 27.8 ± 5.7 (10.7-46). The mean BMI of the patients with graft failure was 27.0 ± 7.5, and that of patients with no graft failure was 27.8 ± 5.7. No statistically significant association was found between BMI and graft failure by Student’s t-test. Four hundred and twenty-two (63.1%) patients were men and 247 (36.9%) were women. Six (75%) patients with graft failure were men and the remaining two (25%) were women. Half of the patients (335 (50.1%)) were married, 210 (31.4%) were single, 13 (1.9%) were widowed, 11 (1.6%) were divorced, and the status of 100 (14.9%) was unknown (Table 1).

**Table 1:** Patients characteristics and their association with graft failure t: The p-value was calculated using Student’s t-test. Other p-values were calculated using Fisher’s exact test.

Variable	Overall	Graft Failure		P-value
		Yes	No	
Age (in years): Mean ± SD (range)	44.4 ± 17.1 (1–86)	43.6 ± 19.9	44.4 ± 17.1	0.900^t^
BMI: Mean ± SD (range)	27.8 ± 5.7 (10.7–46)	27.0 ± 7.5	27.8 ± 5.7	0.712^t^
Gender: n (%)				
Male	422 (63.1)	6 (75)	416 (62.9)	0.717
Female	247 (36.9)	2 (25)	245 (37.1)	
Marital Status: n (%)				
Single	210 (31.4)	3 (37.5)	207 (31.3)	0.680
Married	335 (50.1)	5 (62.5)	330 (49.9)	
Widowed	13 (1.9)	0 (0)	13 (2)	
Divorced	11 (1.6)	0 (0)	11 (1.7)	
Unknown	100 (14.9)	0 (0)	100 (15.1)	

**Figure 1:**
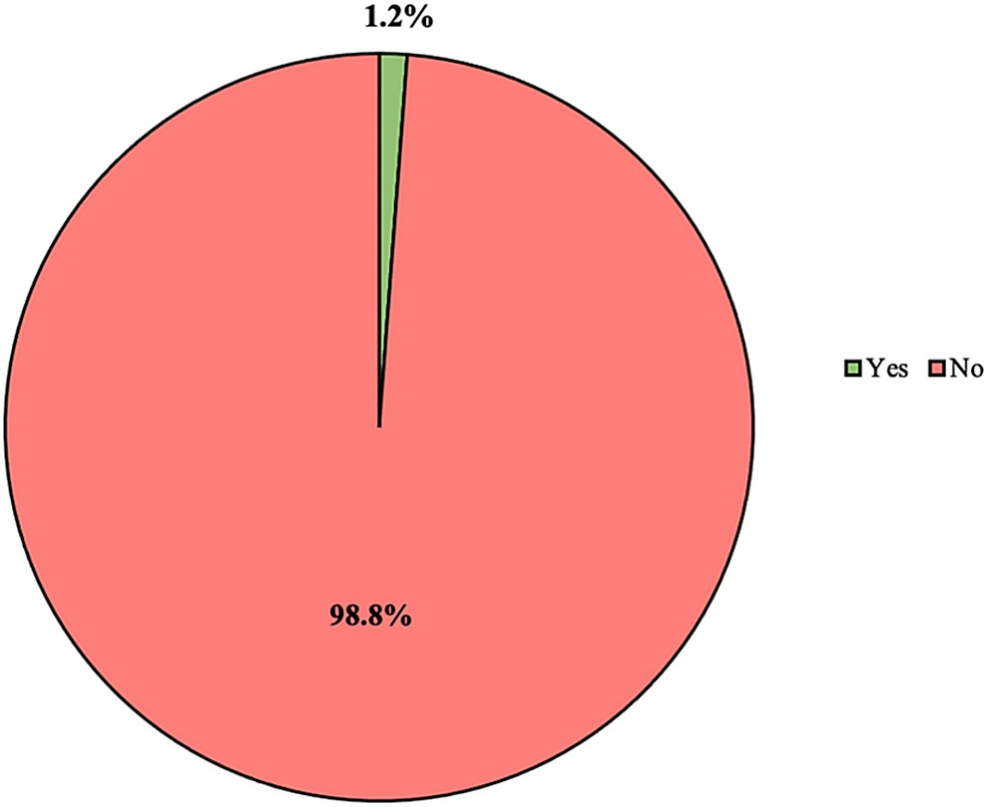
Incidence of graft failure within one year at King Abdulaziz Medical City

The incidence of graft failure within one year at KAMC was 1.2% (eight cases) (Figure 1).

Regarding the CCI and its association with graft failure within one year, 37 (5.5%) patients had MI and 17 (2.5%) had congestive heart failure; no patients with MI or congestive heart failure had graft failure, and no significant association found between MI or congestive heart failure and graft failure (p-value = 1.000 for both). Peripheral vascular disease (PVD) was found in 16 (2.4%) patients, of whom one (12.5%) experienced graft failure; PVD and graft failure were not found to be significantly associated (p-value = 0.177). None of the patients with cerebrovascular accident (CVA; 11 (1.6%) patients), chronic obstructive pulmonary disease (COPD; four (0.6%) patients), connective tissue disease (18 (2.7%) patients), and peptic ulcer disease (PUD; seven (1%) patients) were found to have graft failure; thus, graft failure was not found to be significantly associated with CVA, COPD, connective tissue disease, and PUD (p-value = 1.000 for all parameters).

Concerning liver disease; 24 (3.6%) patients had mild disease, 11 (1.6%) had moderate to severe disease, and the remaining 634 (94.8%) had no liver disease. No patients with graft failure had liver disease, and liver disease and graft failure were not found to be significantly associated (p-value = 1.000). A total of 417 (62.3%) of the patients had no or diet-controlled diabetes, 122 (18.2%) patients had uncomplicated DM, and 130 (19.4%) had end-organ damage. Five (62%) patients with graft failure did not have DM, two (25%) had uncomplicated DM, and one (12.5%) had DM with end-organ damage; DM and graft failure were not significantly associated (p-value = 1.000). Twenty-four (3.4%) patients had localized cancer, one (0.1%) had metastatic cancer, two (0.3%) had leukemia, three (0.4%) had lymphoma, and 644 (96.3%) did not have cancer. Eight (100%) patients with graft failure did not have cancer, and no significant association was found between cancer and graft failure (p-value = 1.000). Five hundred and twenty-one (77.9%) patients were hypertensive and six (75%) of those with graft failure were hypertensive, but no significant association was noted between hypertension and graft failure (p-value = 1.000). Sixteen (2.4%) patients were found to be antibody-positive for hepatitis C virus (HCV); however, none of these patients had graft failure. The mean dialysis duration was 2.83 ± 2.85 years. A total of 645 (96.4%) patients had multiple risk factors, and all patients with graft failure had multiple risk factors (Table 2).

**Table 2:** CCI score and additional comorbidities and their association with graft failure within one year t: The p-value was calculated using Student’s t-test; other p-values were calculated using Fisher’s exact test. CCI = The Charlson Comorbidity Index

Variable	Overall n (%)	Graft Failure		P-value
		Yes (n = 8)	No (n = 661)	
CCI Score				
Myocardial infarction (MI)	37 (5.5)	0 (0)	37 (5.6)	1.000
Congestive heart failure (CHF)	17 (2.5)	0 (0)	17 (2.6)	1.000
Peripheral vascular disease (PVD)	16 (2.4)	1 (12.5)	15 (2.3)	0.177
Cerebrovascular accident (CVA) or transient ischemic attack (TIA)	11 (1.6)	0 (0)	11 (1.7)	1.000
Dementia	0 (0)	0 (0)	0 (0)	-
Chronic obstructive pulmonary disease (COPD)	4 (0.6)	0 (0)	4 (0.6)	1.000
Connective tissue disease	18 (2.7)	0 (0)	18 (2.7)	1.000
Peptic ulcer disease (PUD)	7 (1)	0 (0)	7 (1.1)	1.000
Liver disease:				
None	634 (94.8)	8 (100)	626 (94.7)	1.000
Mild	24 (3.6)	0 (0)	24 (3.6)	
Moderate to severe	11 (1.6)	0 (0)	11 (1.7)	
Diabetes Mellitus (DM):				
None or diet controlled	417 (62.3)	5 (62.5)	412 (62.3)	0.890
Uncomplicated	122 (18.2)	2 (25)	120 (18.2)	
End-organ damage	130 (19.4)	1 (12.5)	129 (19.5)	
Hemiplegia	1 (0.1)	0 (0)	1 (0.2)	1.000
Moderate to severe chronic kidney disease (CKD)	669 (100)	8 (100)	661 (100)	-
Solid tumor/cancer:				
None	644 (96.3)	8 (100)	636 (96.2)	1.000
Localized	24 (3.6)	0 (0)	24 (3.6)	
Metastatic	1 (0.1)	0 (0)	1 (0.2)	
Leukemia	2 (0.3)	0 (0)	2 (0.3)	1.000
Lymphoma	3 (0.4)	0 (0)	3 (0.5)	1.000
Acquired immune deficiency syndrome (AIDS)	0 (0)	0 (0)	0 (0)	-
CCI Score: Mean ± SD	3.35 ± 1.74	3.38 ± 1.19	3.53 ± 1.75	0.800^t^
Other Comorbidities				
Depression	13 (1.9)	0 (0)	13 (2)	1.000
Arthritis	15 (2.2)	0 (0)	15 (2.3)	1.000
Asthma	26 (3.9)	0 (0)	26 (3.9)	1.000
Hypertension	521 (77.9)	6 (75)	515 (77.9)	1.000
Hepatitis C virus (HCV) antibody:				
Positive	16 (2.4)	0 (0)	16 (2.4)	1.000
Negative	653 (97.6)	8 (100)	645 (97.6)	
Dialysis duration (years): Mean ± SD	2.83 ± 2.85	6.50 ± 3.66	2.77 ± 2.80	< 0.001^t^
No. of risk factors:				
Only one risk factor	24 (3.6)	0 (0)	24 (3.6)	1.000
Multiple risk factors	645 (96.4)	8 (100)	637 (96.4)	

Panel-reactive antibody (PRA) was found to be positive in 104 (15.5%) patients via a Luminex class 1 screen. A Luminex class 2 screen showed that 115 (17.2%) patients had a positive PRA, while the remaining 565 (82.8%) had a negative PRA.

The mean serum creatinine level pre-transplantation was 799 ± 300 micromoles per liter (μmol/L), decreasing to 137 ±148 μmol/L at day 7 post-transplantation and reaching 104 ± 74 μmol/L at day 180 post-transplantation. Regarding eGFR, the mean eGFR was 7.3 ± 3.9 mL/min/1.73 m², reaching an average of 69.2 ± 30.2 mL/min/1.73 m² at day 7 post-transplantation and up to 74.9 ± 21.5 mL/min/1.73 m² at day 180 post-transplantation. The mean blood urea nitrogen (BUN) pre-transplantation was 20.5 ± 41.2 mg/dL; at day 7 post-transplantation, the average BUN was 9.2 ± 8.4 mg/dL, decreasing to 6.3 ± 4.6 mg/dL at day 180 post-transplantation (Table 3).

**Table 3:** PRA and creatinine, eGFR, and BUN values PRA = Panel-reactive antibody, eGFR = estimated glomerular filtration rate, Pretx = pre-transplantation, D0 = day of the transplantation, D1 = day 1 post-transplantation, D7 = day 7 post-transplantation, D30 = day 30 post-transplantation, D60 = day 60 post-transplantation, D90 = day 90 post-transplantation, D180 = day 180 post-transplantation

Variable	Categories	N (%)
PRA	Luminex class 1 screen:	
	Positive	104 (15.5)
	Negative	565 (84.5)
	Luminex class 2 screen:	
	Positive	115 (17.2)
	Negative	554 (82.8)
	PRA method:	
	Luminex screen	669 (100)
		Mean ± SD
Creatinine	pretx	799 ± 300
	D0	578 ± 261
	D1	313 ± 206
	D7	137 ± 148
	D30	110 ± 76
	D60	107 ± 69
	D90	106 ± 72
	D180	104 ± 74
eGFR	pretx	7.3 ± 3.9
	D0	11.2 ± 6.6
	D1	27.9 ± 20.8
	D7	69.2 ± 30.2
	D30	70.0 ± 22.8
	D60	70.8 ± 21.9
	D90	72.9 ± 21.7
	D180	74.9 ± 21.5
BUN	pretx	20.5 ± 41.2
	D0	14.9 ± 18.5
	D1	11.3 ± 10.2
	D7	9.2 ± 8.4
	D30	7.3 ± 6.1
	D60	6.9 ± 6.1
	D90	6.6 ± 5.5
	D180	6.3 ± 4.6

Of the patients, 286 (42.8%) had ESRD of unknown etiology, while in 109 (16.3%) patients, the cause of ESRD was diabetic nephropathy. Hypertension was the etiology of ESRD in 100 (14.9%) patients, while 28 (4.2%) patients had IgA nephropathy, seven (4%) were diagnosed with focal segmental glomerulosclerosis (FSGS), 21 (3.1%) were living with polycystic kidney disease, 16 (2.4%) had nephrotic syndrome, 11 (1.6%) had lupus nephritis, 10 (1.5%) had reflux nephropathy, nine (1.3%) had Alport syndrome, seven (1%) were living with neurogenic bladder, six (0.9%) each had atrophic kidney and bilateral hypoplastic kidneys, and 47 (7%) had other etiologies.

The mean age at transplant was 41.2 ± 17.2 years. Concerning the type of surgery, living donor kidney transplantation was performed in about 557 (83.3%) patients, whereas 112 (16.7%) patients underwent deceased donor kidney transplantation. Only six (0.9%) patients suffered complications. Twenty-seven (4%) patients had previous transplantations. Thirty-four (5.1%) suffered from delayed graft function. The mean cold ischemia time was 215 ± 264 minutes. Graft failure within one year was observed in eight (1.2%) patients. Five (0.7%) patients died within one-year post-transplantation (Table 4).

**Table 4:** Surgery information

Variable	N (%)
Indications/diagnosis:	
End-stage renal disease (ESRD) of unknown cause	333 (49.8)
Diabetic nephropathy	109 (16.3)
Hypertension (HTN)	100 (14.9)
IgA nephropathy	28 (4.2)
Focal segmental glomerulosclerosis (FSGS)	27 (4)
Polycystic kidney disease	21 (3.1)
Nephrotic syndrome	16 (2.4)
Lupus nephritis	11 (1.6)
Reflux nephropathy	10 (1.5)
Alport syndrome	9 (1.3)
Neurogenic bladder	7 (1)
Atrophic kidney	6 (0.9)
Bilateral hypoplastic kidneys	6 (0.9)
Age at transplant (years)	41.2 ± 17.2
Type of surgery	
Living donor kidney transplantation	557 (83.3)
Deceased donor kidney transplantation	112 (16.7)
Complications	6 (0.9)
Previous transplant	27 (4)
Delayed graft function	34 (5.1)
Cold ischemia time (minutes)	215 ± 264
Graft failure within 1 year	8 (1.2)
Mortality within 1 year	5 (0.7)

## Discussion

Comorbid conditions affect the outcome of kidney transplantation through a variety of factors. Analyzing comorbid conditions among patients undergoing kidney transplantation may result in a better understanding of etiology and consequences. It may also result in improvements in the treatment strategy, prognosis, and long-term outcomes of kidney transplantation [5,13]. This study aimed at identifying the patient-related risk factors of graft failure following kidney transplant at KAMC, Riyadh, Saudi Arabia.

The mean age of the patients was found to be 44.4 years. Nearly two-thirds (63.1%) of the patients were men. The incidence of graft failure within one year at KAMC was found to be 1.2% (eight cases). This incidence was lower than that reported in a congruent study by Hart et al., in which an incidence of at least 3% was mentioned [14].

Concerning the CCI and its association with graft failure within one year, about 5.5% of the patients had MI and 2.5% had congestive heart failure, but none of these patients had graft failure and no significant association was found between MI or congestive heart failure and graft failure. This result was contradictory to the findings of a study by Morales et al., who found an association between ischemic heart disease and graft failure among the study patients [15]. More than half (62.3%) of the patients had no or diet-controlled diabetes, less than one-fifth (18.2%) of the patients had uncomplicated DM, and 19.4% had end-organ damage. More than half (62%) of those with graft failure did not have DM, one-quarter (25%) had uncomplicated DM, and one (12.5%) had DM with end-organ damage. Thus, DM and graft failure were not significantly associated. However, this result was not in agreement with a parallel study conducted by Taber et al., in which pre-existing DM was found to be significantly associated with graft failure [16]. No significant association was found between cancer and graft failure, consistent with the findings of a study by Kim et al., in which no significant association was noted between cancer and graft failure [17]. More than two-thirds (77.9%) of the patients were hypertensive, and the majority (75%) of those with graft failure were hypertensive, but no significant association was noted between hypertension and graft failure. Similar results were obtained by Weir et al., who also reported a prevalence of hypertension of between 50% and 80% among kidney transplant recipients [18].

The vast majority (96.4%) of the patients had multiple risk factors, and all those with graft failure had multiple risk factors. The mean dialysis duration for the patients was 2.83 years, similar to the findings of a congruent study by Srohmaier et al., in which the median time from the start of dialysis to transplantation was 3.2 years [19]. Regarding eGFR, the mean eGFR was 7.3 mL/min/1.73 m², reaching an average of 69.2 mL/min/1.73 m² at day 7 post-transplantation and up to 74.9 mL/min/1.73 m² at day 180 post-transplantation. The mean BUN pre-transplantation was 20.5 mg/dL, and the average BUN at day 7 post-transplantation was 9.2 mg/dL, decreasing to an average of 6.3 mg/dL at day 180 post-transplantation. The average serum creatinine pre-transplantation was 799 μmol/L, decreasing to an average of 137 μmol/L at day 7 post-transplantation and reaching an average of 104 μmol/L at day 180 post-transplantation. This was found to be consistent with the findings of Morgan et al., who showed a similar course of serum creatinine readings over the same period [20].

The mean age at transplant was 41.2 years; this finding was comparable to that mentioned in Poggio’s study, which reported a mean age of 49.4 years at the time of transplantation [21]. Regarding the type of surgery, living donor kidney transplantation was performed for the vast majority (83.3%) of the patients. Only 0.9% of the patients suffered complications, and 4% of the participants had previous transplantations. Thirty-four (5.1%) participants suffered from delayed graft function. The mortality rate within one year was found to be 0.7%. Less than half (42.8%) of the patients had ESRD of unknown etiology. In about 16.3% of the patients, diabetic nephropathy caused ESRD. Hypertension was found to cause ESRD in about 14.9% of the patients, followed by IgA nephropathy (4.2%) and others. Analogous findings were noted in a study by Hashmi et al., in which diabetic nephropathy was the most frequently reported cause of ESRD [22]. Another study by Banaga et al. found that the most commonly reported etiological cause of ESRD was hypertension [23]. These minor differences could be attributed to different factors, including the study sample and the distribution of diseases.

The limitations of the current study were the fact that the data were gathered from one center, which limited the generalizability of the outcomes. This topic requires more exploration with a larger sample size and should include multiple centers in the region to reach an accurate estimate of the related comorbidities found in patients undergoing renal transplantation.

## Conclusions

Most of the patients were found to have ESRD of unknown etiology and the most frequently reported known risk factor for ESRD and subsequent transplantation was found to be diabetic nephropathy, followed by hypertension. The incidence of graft failure within one year was about 1% lower than that reported in most parallel studies. All patients with graft failure were found to have multiple etiological risk factors.

More effort should be made toward the health education of the general population about the risk factors for ESRD, as knowledge of these etiological factors could result in a reduced incidence of ESRD and hence, the need for transplantation. This could be achieved by encouraging various educational programs, including community events and campaigns, and the distribution of knowledge through social media.
